# Reducing hospital bed use by frail older people: results from a systematic review of the literature

**DOI:** 10.5334/ijic.1148

**Published:** 2013-12-05

**Authors:** Ian Philp, Karen A. Mills, Bhomraj Thanvi, Kris Ghosh, Judith F. Long

**Affiliations:** Hull and East Yorkshire NHS Hospitals Trust, East Riding of Yorkshire, UK; 1 Apley Park Mews, Apley Park, Bridgnorth, UK; Integrated and Community Care, South Warwickshire NHS Foundation Trust, Warwick, UK; Stroke and Medicine for Older People, South Warwickshire NHS Foundation Trust, Warwick, UK; Hull and East Yorkshire NHS Hospitals Trust, East Riding of Yorkshire, UK

**Keywords:** older people, hospital bed use, admissions avoidance, integrated care, systematic review

## Abstract

**Introduction:**

Numerous studies have been conducted in developed countries to evaluate the impact of interventions designed to reduce hospital admissions or length of stay (LOS) amongst frail older people. In this study, we have undertaken a systematic review of the recent international literature (2007-present) to help improve our understanding about the impact of these interventions.

**Methods:**

We systematically searched the following databases: PubMed/Medline, PsycINFO, CINAHL, BioMed Central and Kings Fund library. Studies were limited to publications from the period 2007-present and a total of 514 studies were identified.

**Results:**

A total of 48 studies were included for full review consisting of 11 meta-analyses, 9 systematic reviews, 5 structured literature reviews, 8 randomised controlled trials and 15 other studies. We classified interventions into those which aimed to prevent admission, interventions in hospital, and those which aimed to support early discharge.

**Conclusions:**

Reducing unnecessary use of acute hospital beds by older people requires an integrated approach across hospital and community settings. A stronger evidence base has emerged in recent years about a broad range of interventions which may be effective. Local agencies need to work together to implement these interventions to create a sustainable health care system for older people.

## Background

There is an emerging consensus about the definition of frailty in older people and its association with increased hospital admissions due to falls, confusion and loss of mobility and with increased length of stay once admitted to hospital [1]. Furthermore, there appears to be a frailty phenotype associated with unintentional weight loss, self-reported exhaustion, poor physical activity, slow gait speed and weak grip strength [2]. Policy makers are concerned about the impact of ageing populations and in particular on the increased numbers of frail older people in creating rising demand for acute hospital beds. There is therefore a strong interest in identifying interventions which are effective in reducing avoidable hospital admissions and in reducing the length of stay amongst frail older people.

In this study, we have undertaken a systematic review of the recent international literature (2007-present) to help improve our understanding about the interventions which appear to work and those which have been less successful in reducing hospital bed use.

## Method

We systematically searched the following databases: PubMed / Medline, PsycINFO, CINAHL, BioMed Central and Kings Fund library using the following search filters; older people/frail older people/elderly people/geriatrics and hospital admission rates/hospital length of stay/hospital bed use/early supported discharge/admission avoidance/intermediate care. Studies were limited to publications from the period 2007-present. A total of 514 studies were identified. Following a review of study abstracts, we excluded those which did not include older people or frail older people as the main target population, studies which only included older people with specific long-term conditions and studies of lower quality, for example, single case studies, audit studies and those which did not include a comparison population.

## Results

A total of 48 studies were included for full review consisting of 11 meta-analyses, 9 systematic reviews, 5 structured literature reviews, 8 randomised controlled trials and 15 other studies (6 before and after studies, 6 non-randomised controlled trials, 1 comparator group study, 1 cohort study with case controls and 1 observational cohort study). With only 1 exception [3], evidence from meta-analyses and systematic reviews was classified as high, from literature reviews and randomised controlled trials as medium and that from ‘other’ studies as low.

We assessed the impact of the studies based on the reported findings as follows: Positive - statistically significant positive impact on hospital admissions/readmissions and/or length of stay; Equivocal - some positive but not statistically significant impact; Negative - no impact. We classified interventions into those which aimed to prevent admission (Table 1) interventions in hospital (Table 2), and those which aimed to support early discharge (Table 3).

We found evidence for the effectiveness of care coordination, preventive health checks and care home liaison in the prevention of admission to hospital. Within the hospital setting, there was an evidence for the effectiveness of geriatric assessment units and orthogeriatric units targeting frail older people in reducing the length of stay. For services which linked hospital- and community-based care, including discharge planning, information sharing and rehabilitation services provided in the person's home, there was an evidence of effectiveness in reducing length of stay and preventing readmission to hospital.

There were a series of interventions where there was no evidence of impact on hospital bed use. These included multi-factorial falls prevention services, day hospital services, medication reviews, exercise programmes in the community, nutritional enhancement in hospital and nurse-led transitional care units.

## Discussion

Our search for peer-reviewed publications about interventions for reducing hospital bed use by frail older people published since 2007 revealed a large number of studies. There may be further studies which were not captured by our search terms. As the majority of studies we identified were secondary reviews, our study covers a substantial body of evidence from peer-reviewed research on this topic.

We have found that the evidence base has strengthened for many interventions in hospital and community settings. These include: targeted preventive health checks, care coordination for frail older people, when embedded within integrated health and social care teams, hospital geriatric assessment and orthogeriatric units, community-based rehabilitation services and better integration of acute and post-acute care through discharge planning and joined up information systems.

We have found no evidence to support multi-factorial falls prevention services, community-based medicines reviews, day hospital services, exercise interventions in hospital and nurse-led transitional care, but there were fewer studies of these interventions. It may be that with further development, some of these interventions may prove effective. Studies of association have shown that falls [51], polypharmacy [52], poor nutrition [53–55] and lack of exercise [56] are all associated with increased hospital bed use in older people, so interventions targeted on these areas have the potential to reduce hospital bed use.

Despite huge expectations, telehealth and telecare have not been shown to be effective in the randomised trials. In a recently published randomised trial of telehealth [57] (the Whole Systems Demonstrator telehealth trial), compared with usual care, telehealth was not more effective and did not improve quality of life or psychological outcomes for patients with chronic obstructive pulmonary disease, diabetes or heart failure over 12 months [58]. Reassuringly, no deleterious effects on the service users were noted with the telehealth. Similarly, a cluster randomised trial comparing telecare (as implemented in the Whole Systems Demonstrator trial) with usual care did not show significant reductions in service use over 12 months [59].

Other factors associated with increased bed use include advanced old age [60,61], poor grip strength [62,63], other markers of frailty [64,65], atypical disease presentation [66], multiple co-morbidities [67,68], depression [69,70], cognitive impairment [71–74], poor functional status [75–77], development of pressure sores [78], low socio-economic status [79], lack of family support [80], loneliness [81] and living in a care home [82,83]. These underlying risks are inter-related, which may explain why many of the effective interventions identified in our study were multifaceted.

Effective interventions had common features including anticipatory care targeting older people at risk of adverse outcomes in all settings and well-integrated multidisciplinary practice and inter-agency working. We conclude that services should be developed as a whole system including preventive care, acute hospital care and community care. A shared information system should be created to support patient flow through the system.

## Conclusion

Reducing unnecessary use of acute hospital beds by older people requires an integrated approach across hospital and community settings. A stronger evidence base has emerged in recent years about a broad range of interventions which may be effective. Local agencies need to work together to implement these interventions to create a sustainable health care system for older people.

## Figures and Tables

**Table 1.**
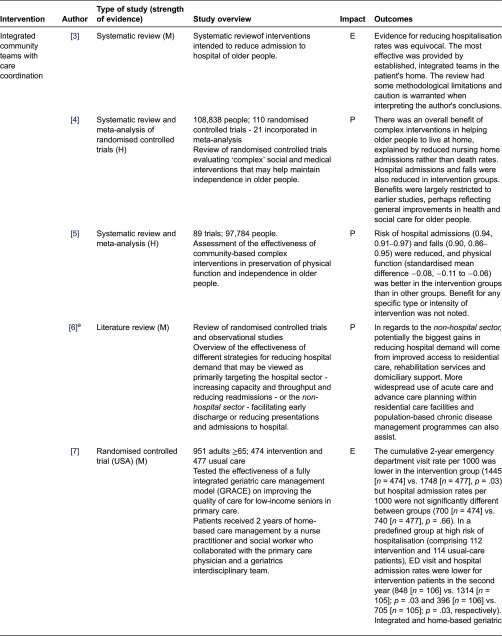

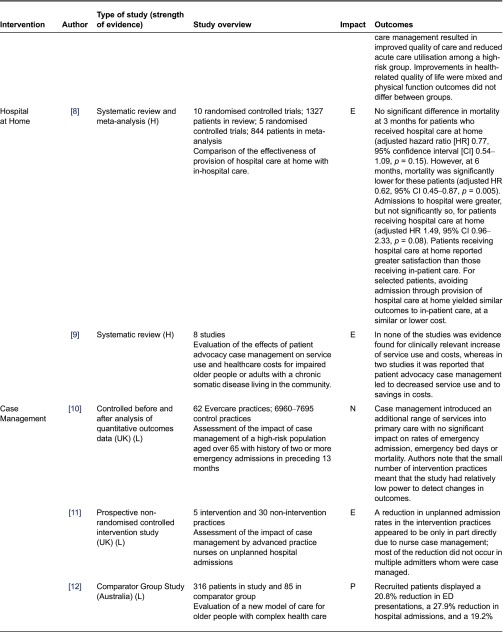

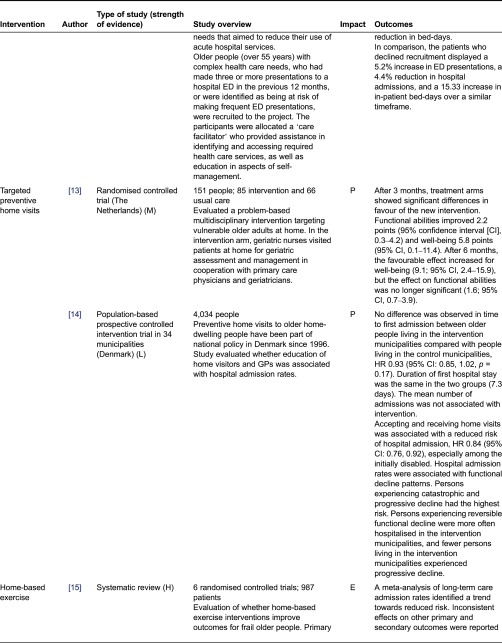

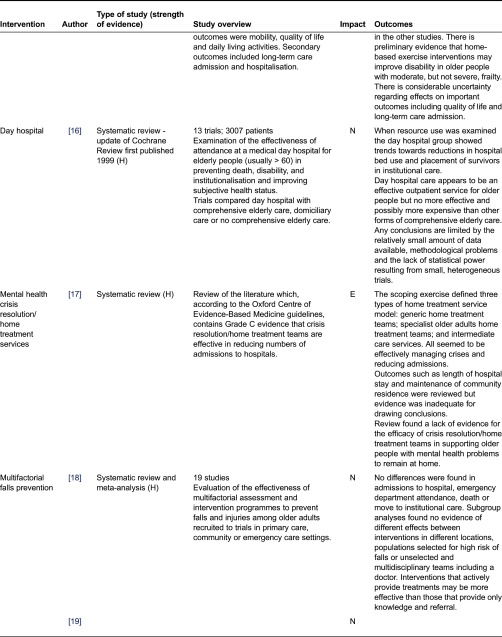

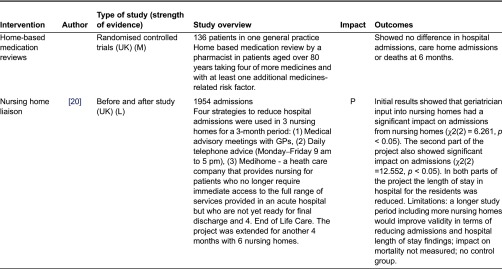
Admission prevention

Impact Key: P = Positive N = Negative E = Equivocal.

Strength of Evidence Key: H = High M = Medium L = Low.

^a^Study included in more than one table.

**Table 2.**
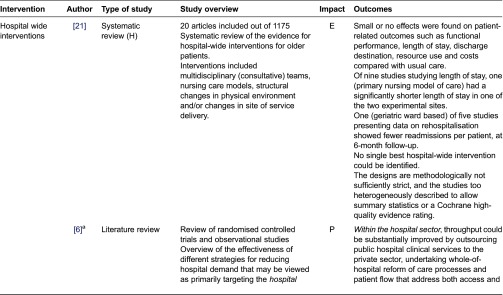

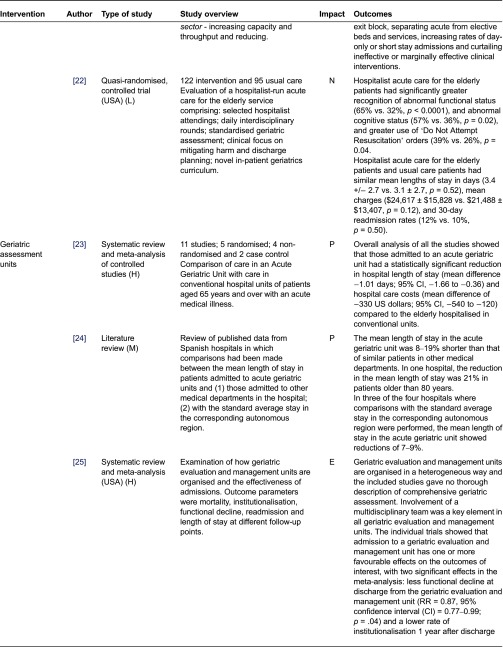

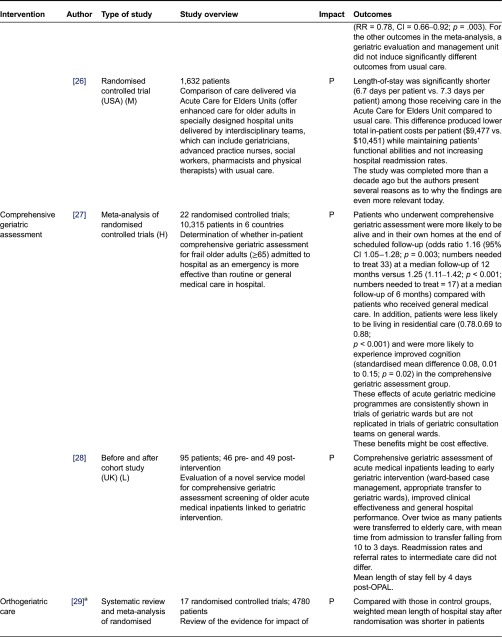

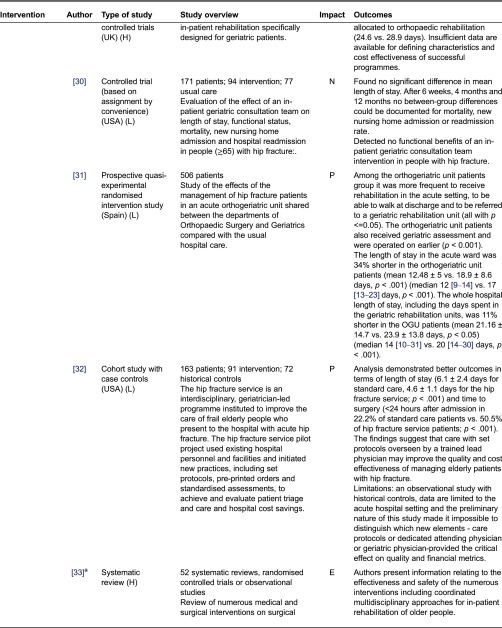

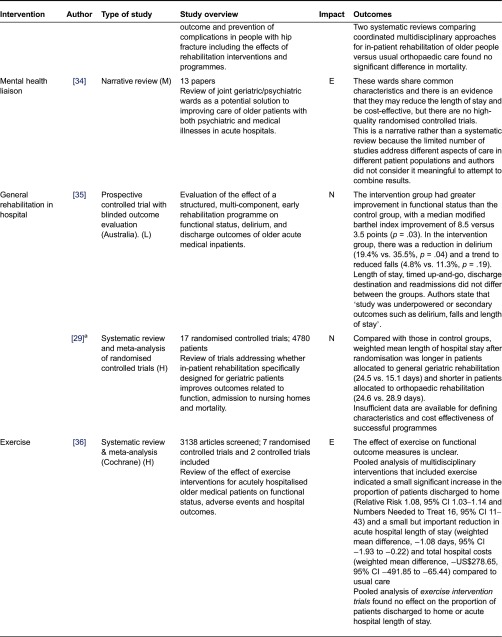

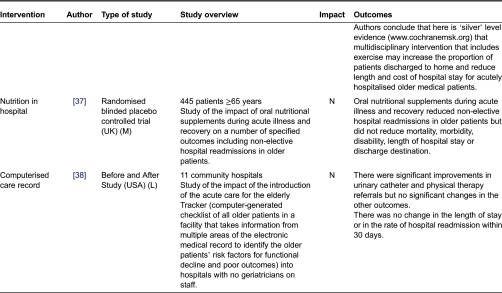
Interventions in hospital

**Table 3.**
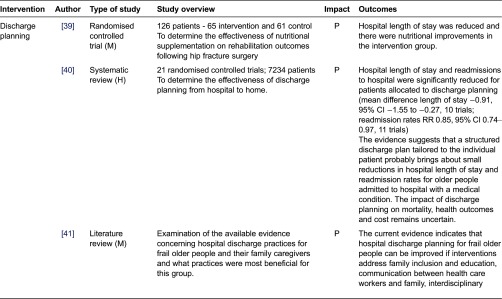

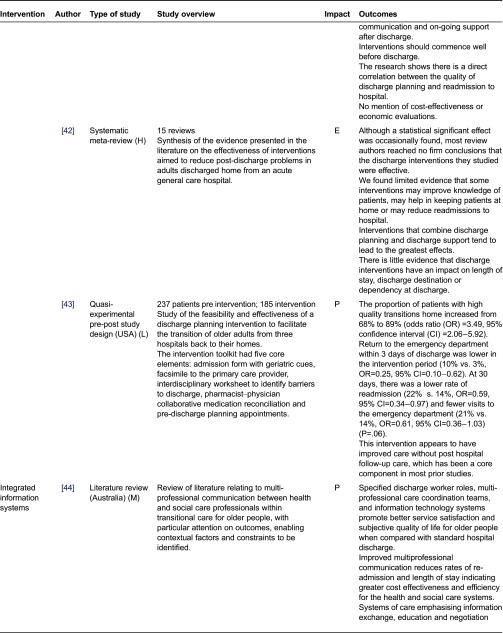

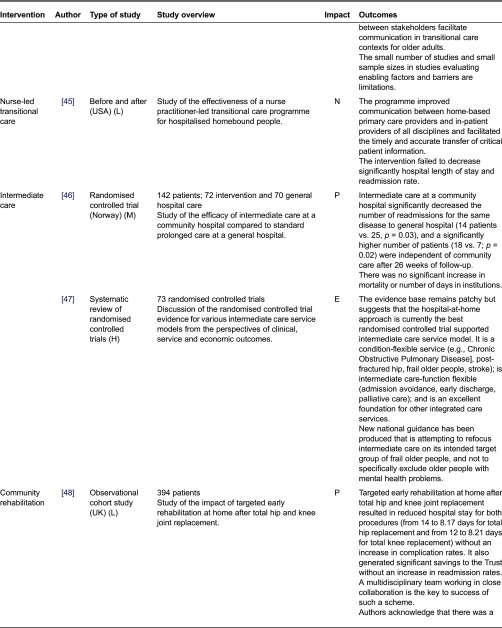

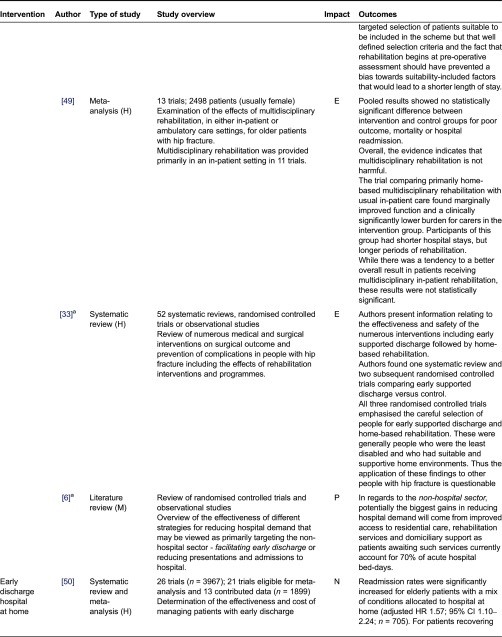

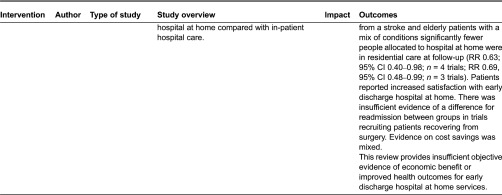
Interventions supporting discharge
